# Rabies Outbreak in an Urban, Unmanaged Cat Colony — Maryland, August 2024

**DOI:** 10.15585/mmwr.mm7431a2

**Published:** 2025-08-21

**Authors:** Sarah Ludmer, David Crum, Ryan Wallace, Jeré Hutson

**Affiliations:** ^1^Division of Workforce Development, Public Health Infrastructure Center, CDC; ^2^Maryland Department of Health, Baltimore, Maryland; ^3^Division of High-Consequence Pathogens and Pathology, CDC.

**Summary**

**What is already known about this topic?**

Cats are the most frequently reported rabid domestic animal in the United States, with approximately 200–300 reports each year.

**What is added by this report?**

In August 2024, identification of a rabid feral cat, initially thought to have potentially exposed 309 persons, led to administration of postexposure prophylaxis (PEP) to three persons.

**What are the implications for public health practice?**

Feral cats pose a rabies risk to local communities. Improved understanding of rabies transmission within cat colonies with subsequent human rabies exposure is needed. Public health engagement is required to educate the public about rabies prevention and health risks associated with feral cat populations and ensure prompt administration of rabies PEP when indicated.

## Abstract

Exposure to a rabid animal is a life-threatening emergency because infection is almost always fatal if rabies postexposure prophylaxis (PEP) is not initiated promptly. In August 2024, the Maryland Department of Health was notified that an aggressive feral cat had been captured and euthanized and had subsequently received a positive test result for rabies. The cat was part of a group of approximately 20 local feral cats and kittens that was receiving no, or little, human care (i.e., an unmanaged cat colony). Cecil County (Maryland) Health Department and the Maryland Department of Health coordinated with Cecil County Animal Services, Cecil County Emergency Services, and CDC to identify and interview persons potentially exposed to the rabid cat, which included a novel use of reverse 911 messaging. Three persons were identified who had been exposed, and all received PEP. No human rabies cases occurred. Three additional cats from the colony were captured and euthanized; their rabies test results were negative. Unmanaged cat colonies pose public health risks, and extensive resources might be required to prevent negative health outcomes. Cat colony management, including activities to maintain high rabies vaccination coverage within colonies, can help to mitigate these risks. A better understanding of urban cat ecology and its role in rabies transmission and human exposure is needed.

## Introduction

Exposure to a rabid animal is a life-threatening emergency; infection is almost always fatal if rabies postexposure prophylaxis (PEP), which includes wound care and vaccination (without or with rabies immunoglobulin, according to indications) is not initiated promptly. In the United States, cats are the most frequently reported rabid domestic animal. Each year, approximately 200–300 cats are reported to have rabies (*1*). In Maryland, feral cats accounted for 10% of all reported rabid animals in 2023.* While rabid cat identification is relatively high among domestic animals, over 90% of animal rabies cases occur among wild animals such as racoons, foxes, bats, and skunks (*1*). Fewer than 10 human rabies deaths are reported each year in the United States.^†^

Feral cats often live in colonies, groups usually comprising female cats and their kittens. Cat colonies can be managed or unmanaged. Managed colonies have a designated caretaker or a community program tending to the colony, providing veterinary services including spaying, neutering, and vaccination. Unmanaged colonies are typically either informally cared for by local residents or receive no human care.

## Investigation and Results

### Identification of Rabid Cat

On August 13, 2024, the Maryland Department of Health (MDH) was notified by the Cecil County (Maryland) Health Department (CCHD) that an aggressive feral cat from an unmanaged urban cat colony was found outside a hotel in northeast Maryland. Clinical signs were first observed on August 8, when the cat became aggressive and bit or scratched two local residents. The cat was captured on August 8 or 9 by local animal control and euthanized; 2 days later, the brain tissue tested positive at the state public health lab for rabies. A kitten from the same colony, which displayed similar behaviors, had been most recently seen by hotel staff members on August 13, suggesting that other cats in the colony might have also been rabid during this period.

### Response Activities

CCHD and MDH coordinated with Cecil County Animal Services, Cecil County Emergency Services, and CDC to identify persons potentially exposed to the rabid cat and provide PEP if indicated. Because cats and other domestic animals can shed rabies virus for up to 10 days before onset of clinical signs (*2*), the potentially infectious period was determined to be July 29–August 13. Investigators initiated trapping activities and identified three groups of potentially exposed persons within this time frame: 1) hotel guests and staff members, 2) persons experiencing homelessness near the hotel, and 3) community members living near the hotel. This activity was reviewed by CDC, deemed not research, and conducted consistent with applicable federal law and CDC policy.^§^

**Hotel Guests.** To facilitate outreach to potentially exposed hotel guests, reservation records for hotel stays that occurred during the cats’ infectious period were reviewed, and the name and contact information for the reservation holder were collected. Investigators collaborated with the rabies points of contact for health departments in U.S. states and Canada for out-of-town hotel guests who might have been exposed. Contacted U.S. states were asked to estimate the human resource investment associated with the response.

Hotel guests who were Maryland residents were contacted via telephone or certified letter by their local health department to determine their rabies exposure risk. Maryland investigators asked a series of questions to ascertain 1) whether exposure had occurred and 2) whether PEP was indicated, including identifying any interaction with the rabid cat; exposure to the animal through bites, scratches, or other interactions; and history of previous rabies vaccination. An email was sent to out-of-state rabies points of contact at the state health department to apprise them of the rabies risk and request that they conduct rabies exposure risk assessments for their residents who had stayed at the hotel. CDC contacted Canadian public health officials to inform them of potential exposures among Canadian residents who had stayed at the hotel.

**Local Residents.** A reverse 911^¶^ message was sent to all Cecil County mobile telephone subscribers to aid in identification of potentially exposed persons who lived in the local area but who had not stayed at the hotel. The message included a description of the situation and advised recipients to contact CCHD immediately if they, or anyone they knew, might have been exposed to cats or kittens from the colony or if they were aware of another animal that might have been exposed.

**Persons Experiencing Homelessness and Walk-In Health Centers.** According to hotel staff members and the CCHD special populations team, which provides community-based support to marginalized community members, at least three persons experiencing homelessness were determined to have been staying near the hotel during the cat’s infectious period. The CCHD special populations team worked to reach as many persons experiencing homelessness in the area as possible. The teams also notified nearby walk-in health centers and advised them to contact CCHD should any patients arrive with complaints of a recent animal bite or scratch. State partners were asked to provide estimates of the time needed to complete their outreach and investigations.

### Identification of Exposed Persons

Review of hotel records identified 309 potentially exposed persons (hotel guests and staff members), including 107 (35%) Maryland residents from 10 local health jurisdictions and 202 (65%) persons from 27 U.S. states and Canada. MDH confirmed that 197 (63.8%) of these persons completed risk assessments. No additional exposures were identified among hotel guests. The two known persons with bite or scratch exposure received PEP** at a local emergency department on August 13.

CCHD received one phone call in response to the reverse 911 message; however, assessment revealed that no rabies exposure had occurred. Trusted community partners contacted two persons experiencing homelessness who were potentially exposed; one of these persons was determined to have been exposed on August 9 and received PEP on August 18. Thus, a total of three exposed persons were identified. None of the three persons have developed signs consistent with rabies to date. Among 29 jurisdictions involved, 17 (58.6%) provided personnel time estimates, totaling 450 hours.

## Additional Public Health Response

Local officials determined that the rabid cat was part of an unmanaged colony of approximately 20 cats and kittens. Information from local residents indicated that community members were sporadically providing food or shelter to local feral cats and kittens. Thus, additional animal rabies cases among this colony might have occurred but were not reported.

Due to the concern for additional cats in the colony being rabid, Cecil County Animal Services assisted in the capture and testing of cats in the colony, although the size of the colony was not known. Three additional feral cats from the colony were captured within 3 weeks of identification of the index rabid cat. All three were euthanized and tested for rabies, and their test results were negative. In response to the reverse 911 message, residents living near the hotel reported frequently seeing and feeding feral cats in their area and at a nearby farm, providing insight into the potential size of the cat colony and what type of human care the cats received. The farm owners were contacted, via state dairy regulatory health personnel familiar to the farm owners, to determine whether residents or animals at the farm had been exposed and alert the owners to the situation; no exposures were identified.

## Discussion

Each year, approximately 200–300 cats are reported to have rabies in the United States, making cats the most frequently reported rabid domestic animal (*1*). Feral cats are more likely to interact with wildlife that are reservoirs for rabies and are less likely than are owned pet cats to receive veterinary care. Therefore, feral cats pose a higher risk for human exposure to rabies than wildlife, given the higher likelihood that humans will approach cats (*3*,*4*). CDC estimates that PEP treatment associated with exposure to rabid or potentially rabid cats costs approximately $33 million each year (*3*,*5*).

In Maryland, 109 rabid cats have been reported since 2019. The CDC national rabies surveillance program, which receives reports of human and animal rabies cases from 54 state and territorial jurisdictions, does not track ownership status of cats (*1*). However, news reports and state records indicate that a substantial proportion of rabid cats are feral or cats in managed, urban colonies. Although cat rabies is a recognized problem in the United States, mass exposure events that necessitate large-scale, multistate contact tracing measures are rarely described in the literature. Improved understanding of urban cat ecology and the role of cats in rabies transmission and human exposure events is needed.

When cat colonies are managed, caretakers often attempt to maintain high rabies vaccination coverage and use sterilization programs to facilitate a natural phaseout of the colony (*6*). However, unmanaged cat colonies, such as the one described in this report, can pose public health risks and require extensive resources to prevent adverse human and animal health outcomes. These outcomes can persist indefinitely if natural breeding continues. Persons living in the vicinity of and engaging with the cat colony described in this report were at risk for bites, scratches, and rabies exposure. However, this interaction with the feral cat population also represents an opportunity to engage community members in a managed cat colony program, which could facilitate implementation of recommended strategies including spaying, neutering, and vaccination (*7*). Many jurisdictions have organizations or resources that provide guidance on proper cat colony management.

Outbreaks of deadly pathogens in urban environments potentially expose several population groups; this investigation required multiple outreach strategies, including accessing the international health regulations notification system,^††^ special populations health care teams, trusted farming partners, and reverse 911 systems. Responses to rabid animal exposures also require substantial time from public health responders. Reverse 911 messaging helped provide public health responders with additional details related to the cat colony and additional potentially exposed groups, although it did not result in the identification of any additional exposed persons. Use of existing trusted community outreach partners and the commitment of CCHD staff members were crucial to ensuring that exposed persons, particularly those experiencing homelessness, received PEP.

### Limitations

The findings in this report are subject to at least two limitations. First, not all states reported the total number of residents they had to reach to complete risk assessments or the results of those activities. Second, the human resource investment could only be estimated because not all states reported time estimates to MDH. Thus, both measures in this investigation are likely underestimated.

### Implications for Public Health Practice

The U.S. feral cat population poses an ongoing risk to human health (*3*). Increased public education regarding the health risks posed by unmanaged cat colonies^§§^ might reduce potential human exposure to rabies and other diseases.

## Footnotes

*
Laboratory Confirmed Animal Rabies in Maryland, 2023

†
Rabies | Animal Rabies Surveillance in the U.S. | CDC

§ 45 C.F.R. part 46, 21 C.F.R. part 56; 42 U.S.C. Sect. 241(d); 5 U.S.C. Sect. 552a; 44 U.S.C. Sect. 3501 et seq.

¶ Reverse 911 messaging is a system used by emergency responders to send out alerts to residents in a specific geographic area by text message.

** PEP includes wound cleansing, and depending on previous vaccination status, human rabies immune globulin, and a series of doses of rabies vaccine. PEP should be administered as soon as possible after exposure and in accordance with CDC guidelines. Rabies Post-exposure Prophylaxis Guidelines | CDC

††
International Health Regulations (2005) | Third Edition | World Health Organization

§§
Free-roaming Abandoned and Feral Cats | American Veterinary Medical Association
